# Patient advocacy from the clinical nurses' viewpoint: a qualitative study

**Published:** 2016-06-11

**Authors:** Shirmohammad Davoodvand, Abbas Abbaszadeh, Fazlollah Ahmadi

**Affiliations:** 1 PhD Candidate in Nursing, Razi Nursing and Midwifery College, Kerman University of Medical Sciences, Kerman, Iran;; 2 Professor, Faculty of Nursing and Midwifery, Shahid Beheshti University of Medical Sciences, Tehran, Iran;; 3 Professor, Nursing Department, Faculty of Medical Sciences, Tarbiat Modares University, Tehran, Iran.

**Keywords:** *Clinical nurse*, *Patient advocacy*, *Iran*, *Qualitative research*

## Abstract

One of the advanced nursing care procedures emphasized by nursing organizations around the world is patient or nursing advocacy. In addition to illustrating the professional power of nursing, it helps to provide effective nursing care. The aim of the present study was to explain the concept of patient advocacy from the perspective of Iranian clinical nurses.

This was a qualitative study that examined the viewpoint and experiences of 15 clinical nurses regarding patient advocacy in nursing. The nurses worked in intensive care units (ICUs), coronary care units (CCUs), and emergency units. The study participants were selected via purposeful sampling. The data was collected through semi-structured interviews and analyzed using content analysis.

Data analysis showed that patient advocacy consisted of the two themes of empathy with the patient (including understanding, being sympathetic with, and feeling close to the patient) and protecting the patients (including patient care, prioritization of patients’ health, commitment to the completion of the care process, and protection of patients' rights).

The results of this study suggest that nurses must be empathetic toward and protective of their patients. The results of the present study can be used in health care delivery, nursing education, and nursing management and planning systems to help nurses accomplish their important role as patient advocates. It is necessary to further study the connections between patient advocacy and empathy.

## Introduction

Patient advocacy in nursing is a relatively modern idea (1), but its first movements originated in Florence Nightingale’s era (2). It is of such importance that it has entered the moral codes of nursing institutions (1, 3). The need for justice is among the basic human needs (4) and nurses, more than anyone else, are in contact with patients and their problems (5); therefore, they can provide justice for the patients better than anyone else (6). Nurses are the first advocates of patients (7), and are the link between the patient and the health care system (8).Patient advocacy is one of the extremely important roles of the nurses (9-12). 

The patient or client is vulnerable and has experienced varying degrees of damage (13). Therefore, many opportunities arise in nursing for the enforcement of patient advocacy, which has turned the nursing profession into the most reliable profession regarding patient advocacy. Through the appropriate performance of this role, the trust and respect of the community toward the nurses will increase (7). Nevertheless, even when they have effectively performed their role, complications such as fear, anger, frustration, hopelessness, and a sense of separation from their peers are experienced (1, 9). However, effective advocacy improves the quality of patient care and enriches the nursing profession. Thus, the failure to play this role effectively may detract from the richness of this profession (14) and result in nurses leaving their profession (15). 

Advocacy is generally described as defending the rights and property of others (3). In nursing, it has been defined as being a patient representative, defending the patient’s rights and universal rights, protecting the interests of the patient, contributing to decision-making and supporting the patient’s decisions (3, 11, 16), ethical-centered skills for the ‘professional self’(17), and ‘being a voice for the vulnerable (3, 18). Negarandeh et al. defined the dimensions of patient advocacy in Iranian nurses as informing and educating, valuing and respecting, and physical, emotional, and financial support, protecting and representing the patient, and continuity of care (13). However, providing a single definition for the term is difficult (8, 11).

Today, patient advocacy has taken a wider range of dimensions. For example, Ware et al. stated that protecting patients against unethical and illegal acts was only a part of patient advocacy (12). Mahlin stated that although supporting the patient is a major goal, the broader problems of patient advocacy cannot be resolved through this method, and the patient’s advocate should address the systematic problems of care and administrative institutions (19). In support of this claim, Maryland and Gonzalez stated that nurses, in addition to within hospitals, should support patients and their families in other social environments including economic, educational and research, healthcare delivery, and legislative environments, regarding their access to health care, cost control, and health care quality (8). Protection of clients in clinical trials (20), supporting of organ donation volunteers, and protection of the fundamental rights and welfare of patients are also added to this category (16).

Patient advocacy is an ideal in the nursing practice (21). It is reliant upon many factors, including social relationships, human interactions (22), and moral distress and its side effects. However, many aspects of this concept have not been identified (23). Many studies have referred to the failure to define and explain the concept of nursing advocacy and their results were not in agreement (8, 10). These ambiguous interpretations of patient advocacy impose a number of problems on the nursing practice (21). Considering nurses’ lack of knowledge on patient advocacy in nursing and its irreparable consequences, it is necessary to train nurses on patient advocacy (9, 11). 

In addition, few Iranian studies have addressed this issue. Jafari Manesh et al. in their descriptive-comparative study found that this perception was higher in the patents than the nurses and higher in the nurses than the physicians, but they did not address the topic of patient advocacy itself (24). Negarandeh et al., through the grounded theory, explored the dimensions of patient advocacy in Iranian nurses (13). So the present study is part of a greater qualitative study and its results differed in some ways from the results of previous studies especially in terms of empathy. Thus, this topic requires further qualitative exploration. The aim of this study was to better clarify nursing advocacy among Iranian nurses through a qualitative study. 

## Method

The present text is a part of a larger qualitative study and this portion of data was analyzed using a conventional content analysis approach to explore clinical nurses’ experiences and perspectives of patient advocacy. Qualitative research is suitable for studies on relatively new areas of knowledge (25). In qualitative researches, content analysis is largely applied today to the interpretation of textual data (26) that tend to review less well-known phenomena in their natural environment based on individuals’ views and experiences (27). Patient advocacy is a very complex topic in the health care system (9). Patient advocacy, like other ethical issues, is related to the socio-cultural context. Therefore, the conventional content analysis approach was used in the present article to study patient advocacy in nursing.

**Participants**

15 nurses selected through purposeful sampling to participate in the study.

**Data collection and analysis**

A total of 18 semi-structured face-to-face interviews were conducted from June 2012 until June 2013 to collect the data; 3 interviews were repeated. The duration of the interviews was 25 to 75 minutes with an average of 53.3 minutes. The location and the duration of the interviews were selected by the participants. The primary research questions included: “Can you please explain your relationship with the patient as a nurse?” or “What are your responsibilities toward the patient as a nurse?” Exploratory questions were also asked during the interviews to obtain the participants' experiences and opinions and to clarify their responses. Data collection and analysis were carried out simultaneously. The interviews were recorded and, in order to obtain a general understanding, were studied at least 3 times. The recordings were transcribed verbatim. The obtained data were analyzed according to the following steps (28). 

1. In order to gain a general understanding of them, the interviews were studied several times, and then, they were transcribed verbatim.

2. The interview texts were divided into compact semantic units. 

3. The compact semantic units were converted into abstract terms and assigned a specific code. 

4. The emerged codes were categorized based on their differences and similarities into subclasses and classes.

5. The themes were extracted from the interviews.

The research team found no new information of relevance to their study in the data in the 15^th^ interview. This is interpreted as data saturation in qualitative research, and thus, no more interviews were needed (29).

**Trustworthiness**

To assess the study’s trustworthiness, Lincoln and Guba’s Evaluative Criteria and authenticity were used (30). Lincoln and Guba’s Evaluative Criteria consist of 4 criteria including: 

A-Credibility: It shows that the identification and introduction of research participants are accurate.

B-Dependability: It shows that data remain stable over time and under different conditions.

C-Conformability: It shows objectivity, that is, the potential for congruence between different independent individuals on the accuracy, relevance, or meaning of the data.

D-Transferability: It shows the potential to generalize the findings of the study (29).

Authenticity is an additional criterion that shows the extent to which researchers indicate a range of realities fairly and faithfully (31). 

Due to the prolonged and continuous engagement of the researcher with the data, the participants, and member checking, the credibility of the study increased. For this purpose, the researcher gave a typed summery of the interviews to the participants, so they could confirm his interpretations. In order to meet the confirmability of the data, peer check was used. The researcher first coded and classified each interview, and then, presented these classifications and codes to other members of the research team for evaluation. The codes which were not agreed upon were discussed until achieving clarification and consensus. To control the dependability of the data, the researcher retained the preliminary data, codes, categories, and themes. To achieve transferability or stability of the results, sampling was carried out with great variety to contribute to the credibility of data. 

**Ethical considerations**

This study and its ethical considerations were approved by and the necessary permits were obtained from the Research Deputy of Kerman University of Medical Sciences, Kerman, Iran. All the participants were informed of the nature, purpose, and method of the study, the researchers’ tasks, their rights, and possible risks (this was a safe study) by the first author. The confidentiality of the participants’ statements, and their right to choose to continue or leave the study was emphasized by the research team. All participants consented to the recording of the interviews, and after receiving the necessary information, they signed an informed consent form.

## Results

In the present study, 15clinical nurses with an average work experience of 8 years and 3 months and mean age of 32.25 years were selected from selected wards suitable to the larger study (Table 1). The participants were selected from educational and non-educational hospitals in different provinces of Iran to gain an adequate variation in experiences and perspectives of nurses regarding patient advocacy in nursing. 

**Table 1:** Characteristics of the study participants

**Characteristics**		**Number**
Gender	Male	3
	Female	12
Marital status	Married	7
	Single	8
Education level	Bachelor	14
	Master	1
Working unit	Coronary care unit )CCU)	7
	Intensive care unit (ICU)	5
	Emergency unit	3
Position	Nurse	8
	Head nurse	3
	Supervisor	3
	Nursing manager	1

Interesting results were obtained from content analysis. According to the participants’ statements, patient advocacy included the two themes of empathy with the patient and protecting the patient (Table 2). 

**Table 2:** Results of themes and categories

**Theme**	**Categories**
Empathy with the patient	Understanding the patient's condition
	Showing compassion
	Feeling close to the patient
Protecting the patient	Taking care of the patient
	Being a patron to the patient
	Commitment to completing the care period
	Protecting patient rights

*1. Empathy with patients*

1-1Understanding patients’ conditions: 

Understanding is a psychological relationship between the individual who knows and what they know. It is a process to gain knowledge on an abstract or physical object that in our study is the patient and his/her condition. Nurses’ understanding of the conditions and expectations of patients can improve the nurse-patient relationship. This relationship presented itself as patient advocacy in the present study. The nurses in this study tried to defend their patients, because one day they might become a patient and require care themselves. 

Participant number 10 stated: [*When a patient walks into the hospital, he/she must have a problem. His/her problem is understandable for me. I know that if he/she did not have a problem then he/she would not have come to the hospital. I put myself in his/her shoes and try to help him/her in any way that I can. I do not have any problems with the patients*.]

Participant number 4 stated: [*Each patient has his/her own expectations when he/she comes to the hospital. In the same circumstances, we, ourselves, would want a favorable healthcare service. We would expect an accurate diagnosis from the physician. We would want good healthcare services and would expect to see results*.]

1-2* Showing compassion***:**

Compassion, as an outstanding human characteristic, is a response to the suffering of others and the cause of helping them. In fact, compassion is synonymous with co-suffering. Humans cannot simply overlook the problems of others. They are affected by the suffering and pain of one another, seek solutions to alleviate the pain, and become patrons to others. 

As an example, participant number 3 said: [*I feel sorry when I see that the patient does not have anybody to look after her/him. I cannot stand seeing patients suffer. They feel vulnerable. I try my best to help them*.]

1-3* Feeling close to the patient:*

This theme is defined as the sense of unity with others. This sense does not depend on the conditions of others. It exists in pain or pleasure, but is more common in pain. Humans feel close to their fellow men and perceive their problems as their own. They try to share their sad and happy moments. Nevertheless, this feeling is stronger toward children and their immediate family members, and is heightened during illnesses. Nurses who have this feeling of closeness tend to defend their patients as they would defend themselves. Participant number 15 stated: [*I feel closer to some of the patients, for example, in the Thalassemia unit, especially children who refer to receive blood. I become friends with some of their families. The Thalassemia unit is the second home of these children*.]

Participant number 1 declared: [*When I see a patient, I imagine him/her as being my relative. I help him/her in any way that I would help my own family. I do not withhold anything from the patients*.]

*2. Protecting the patient*

2-1 Taking care of patients*:*

This phrase means caring for the patient in general. It is caution toward others with the aim to prevent individuals from being harmed. One of the main tasks of nurses is to protect patients against injuries and possible risks. These risks may be physical, mental, deliberate, inadvertent, or due to insufficient treatment or incorrect care. Given that disease reduces individuals’ defensive strength, patients need someone to protect them against these threats. Most nurses have experienced this responsibility as a patient advocate. 

Participant number 2 described this issue more objectively: [*We should be careful that the patient does not fall from the bed, protect him/her against any injuries in the ward or against anyone coming to the hospital to hurt him/her, and to control the medication to avoid any further harm*.]

Participant number 7 in confirming the above statements said: [*We must monitor the patient for any side effects of the medication that is being used. The patient is here to get better not to get worse. Sometimes the patient shows reactions to the blood he/she is taking, and we must intervene immediately to prevent any damage to his/her kidneys, and breathing*.]

2-2* Prioritizing patients’ health:*

While working at hospitals, different problems may occur for the nurses. These problems may include family or career issues, interactions with co-workers, or any other problem, and cause the nurses to prioritize their duties. In this case, most of the nurses participating in this study have pronounced the health of their patients as their first priority. Participant number 5 described this skill as: [*When I find that something is not in the best interest of the patient (for example, the behavior of a colleague, or the treatment method or the care being provided by my colleague), I do not think whether my colleague will get upset with me. My colleague’s feelings are my second priority. My patient’s health is the most important matter for me in my working environment. As hard as it may be, I try to only focus on my patient before entering the unit*.]

2-3* Commitment to the completion of the care period:*

By selecting this label for the category, we purposed to address the topic of follow-up in nursing. Patient follow-up is a part of nursing care, and without it, nursing care remains incomplete. Nurses plan to improve their patients’ health and expect to follow this plan until it is finished. According to the participants, the completion of an appropriate plan brings peace of mind to nurses. Actions that interrupt the process cause stress for nurses. This mindset prevents any harm coming to the patients. 

Participant number 14 stated: [*The nurse should provide care as soon as the patient is admitted. We are nurses and we should perform our duties and follow our plans under any circumstances. Even if the patient is in his/her final days of life, it is our duty to take care of him/her. We should provide our services until the patient is discharged or has died*.]

*2-4 Defending patients’ rights:*

Rights are fundamental normative rules that determine what is permitted and what is not. This is a part of the protection of patients from any harm. Every human being has a right. Nurses care for individuals who are unable to defend their rights, or do not know their rights and need help in this regard. Nurses also help the patients by defending and informing them of their rights. 

In regards to her colleagues’ violation of patient’s rights, participant number 6 stated: [*The head nurse follows up with a patient if she feels the patient is not provided with proper care. For example, a patient was being transferred to the emergency unit, but the crowded environment of that unit was harmful to him. The head nurse insisted on transferring the patient to a calmer and more disciplined unit. I would do the same if the welfare of the patient is in danger*.]

Participant number 15 expressed a more subtle point: [*Sometimes the medication of a patient is used for another patient. It is very important for me to replace whatever has been taken from a patient, even if it is a tablet.*]

## Discussion

The aim of patient advocacy, as a fundamental aspect of nursing care, is to provide high quality health care and protect the rights of the clients (32). Nevertheless, some factors, such as the lack of competency and recourses (10), burn out, professional suffering, and lack of dedication to nursing (11) hinder achievement of these goals. On the other hands, these factors place the clients at risk, which increases the importance of patient advocacy.

Considering these barriers, in the present study, patient advocacy in nursing consisted of two themes of empathy with the patient and protecting the patient. These findings have some similarities and differences to other studies that have been conducted in this field. Patients experience different degrees of vulnerability (10). Moreover, Young stated that “all elements of the patient advocacy are consistent with protecting the patient from the harm” (26). Therefore, protection is a key element of patient advocacy. Regarding children, this protection includes protection against child abuse (33, 34).

The nurses participating in this study also suggested that protection forms the predominant part of patient advocacy in nursing. In nursing, however, many internal and external risks threaten a patient's health care environment. The origin of these risks is the disease and the patients’ inability, lack of sufficient defensive power, and lack of sufficient knowledge about the disease, care, and treatment of the disease, and the treatment environment. There are also dangers which threaten the patients’ rights at different levels. The participants of this study had committed themselves to protecting the patients. Patients’ rights and health status, and the nature of the nursing profession were the most important factors that nurses stated as reason for protecting the patient.

Negarandeh et al. have stated that protecting the patient is one of the key factors of patient advocacy, and nurses, as the patient advocate, are responsible for protecting the patient against inadequate health care provided by other healthcare team members (13). Their study was a great study that supports the present study results in patient protection.

Patients often complain about the lack of appropriate verbal communication with the nurses. They choose nurses who establish a close relationship with them as their advocates (35). Empathy is the ability to define the unique situation of others (36), and also an inseparable part of the nurse-patient relationship (37). Bikker et al. found that empathic patient-centered care is a high quality outcome in the health care system and patients constantly measure and score nurses’ empathy and humanistic behavior (38). By responding with empathy to the patient and his/her family, the nurse can help them adapt to their problem, this outcome is in line with patient protection (39). Ferri et al. in an analytical cross-sectional study reported that empathy toward patients has a negative relation with nursing burnout and diminishes the course of the disease (40). Considering these results, which support our findings, it can be stated that empathy is an implicit component of patient advocacy.

Empathy is a personal matter which takes on a more professional aspect in nursing. Patient advocacy is a developed and distinct form of communication between nurses and patients which has been illustrated in the comments of the study participants. From the perspective of the participants, patients require by their side individuals with whom they can share their problems, individuals who understand them and help them. Nurses, like other individuals in these situations, want to gain closeness to others and collaborate with them. Since nurses have been trained for these situations, they try to approach patients in different situations in order to defend their rights with more power and a better understanding of the patient. A common concern is then formed among patients and nurses. It stimulates nurses’ sense of compassion when faced with the suffering and helplessness of their counter parts and increases their willingness to help the patients. A feeling of empathy is formed between nurses and patients as part of patient advocacy. 

Previous studies have not provided any support regarding empathy and its relationship with patient advocacy, but the common elements of empathy, communication, and advocacy cannot be denied. For example, a study in 2015 reported that individuals with higher capacity for empathy can more easily understand and accept the perspectives of others (37).

Tomaschewski Barlem et al. stated that patient advocacy provided by nurses is based on their personal values and professional skills (9). The study by Jafari Manesh et al. (24) differs from our study methodologically and cannot be used to support or contradict our findings. The findings of Negarandeh et al. (13) do not support our findings on empathy. In the Iranian culture, almost everything, including ethics and values, is influenced by religion. Thus, personal value in this culture refers to religious beliefs. Protection of the vulnerable and doing all that is in your power for others is ordered in Islam. However, the data presented in the current text did not indicate this subject explicitly. 

It seems that empathy in patient advocacy in nursing is a relatively new issue that is largely dependent on the social context of the Iranian society, including religious background, and perhaps more specific studies can confirm or repudiate this matter.

## Conclusion

Patient advocacy is a social issue which can be evaluated from personal and professional aspects. In this study, patient advocacy in nursing included the two themes of empathy with patients and protecting patients. Protection of patients in previous studies has been repeatedly defined as an important component of patient advocacy. Nevertheless, empathy with the patient is a relatively new idea and it seems that a more thorough study on this topic can help the better understanding of this relationship. The results of this study can be used in the development of nursing students and novice nurses, retraining of employed nurses, and sensitizing of nursing managers and planners and other related occupations to the improvement of nurses’ performance, reduction of the adverse effects of patient advocacy, and promotion of the health of the society. It is suggested that further studies be conducted on the relationship between empathy and patient advocacy. Future studies may be performed on the effect of religion on patient advocacy, especially in religious societies. 
